# Medical Schools

**Published:** 1835

**Authors:** 


### II. — MEDICAL SCHOOLS.

That our readers may have a full and authentic account of the recent exertions of the profession, to reorganize and improve the condition of the Medical College of Ohio, of the total failure which has occurred, and of the origin of a new school, under the auspices of the Cincinnati College, we have added to this number a supplement or *extra-limites* of 35 pages, to which we beg leave to refer all who are interested, reminding them, that the number contains, independently of those, the usual number of pages.

